# Association between the Severity of Influenza A(H7N9) Virus Infections and Length of the Incubation Period

**DOI:** 10.1371/journal.pone.0148506

**Published:** 2016-02-17

**Authors:** Victor Virlogeux, Juan Yang, Vicky J. Fang, Luzhao Feng, Tim K. Tsang, Hui Jiang, Peng Wu, Jiandong Zheng, Eric H. Y. Lau, Ying Qin, Zhibin Peng, J. S. Malik Peiris, Hongjie Yu, Benjamin J. Cowling

**Affiliations:** 1 Department of Biology, Ecole Normale Supérieure de Lyon, 15 parvis René Descartes, 69007 Lyon, France; 2 WHO Collaborating Centre for Infectious Disease Epidemiology and Control, School of Public Health, Li Ka Shing Faculty of Medicine, The University of Hong Kong, 21 Sassoon Road, Hong Kong Special Administrative Region, China; 3 Division of Infectious Disease, Key Laboratory of Surveillance and Early-Warning on Infectious Disease, Chinese Center for Disease Control and Prevention, 155# Changbai Road, Beijing, 102206, China; Fondazione Bruno Kessler, ITALY

## Abstract

**Background:**

In early 2013, a novel avian-origin influenza A(H7N9) virus emerged in China, and has caused sporadic human infections. The incubation period is the delay from infection until onset of symptoms, and varies from person to person. Few previous studies have examined whether the duration of the incubation period correlates with subsequent disease severity.

**Methods and Findings:**

We analyzed data of period of exposure on 395 human cases of laboratory-confirmed influenza A(H7N9) virus infection in China in a Bayesian framework using a Weibull distribution. We found a longer incubation period for the 173 fatal cases with a mean of 3.7 days (95% credibility interval, CrI: 3.4–4.1), compared to a mean of 3.3 days (95% CrI: 2.9–3.6) for the 222 non-fatal cases, and the difference in means was marginally significant at 0.47 days (95% CrI: -0.04, 0.99). There was a statistically significant correlation between a longer incubation period and an increased risk of death after adjustment for age, sex, geographical location and underlying medical conditions (adjusted odds ratio 1.70 per day increase in incubation period; 95% credibility interval 1.47–1.97).

**Conclusions:**

We found a significant association between a longer incubation period and a greater risk of death among human H7N9 cases. The underlying biological mechanisms leading to this association deserve further exploration.

## Introduction

The incubation period of an infectious disease, which is defined as the time between infection and onset of symptoms, is an important biological parameter with relevance to prevention and control of an epidemic. In early 2013, a novel avian-origin influenza A(H7N9) virus emerged in China [1]. By 15 June 2015, 655 laboratory-confirmed cases were reported in mainland China. The mean incubation period of human infections was around 3.4 days [2], similar to the incubation period for human infections with influenza A(H5N1) [3], and longer than the incubation period for human infections with seasonal influenza viruses [4].

In previous studies, we reported that fatal cases of Severe Acute Respiratory Syndrome (SARS) coronavirus and Middle East Respiratory Syndrome (MERS) coronavirus infections had shorter incubation periods when compared with non-fatal cases [5,6]. The relationship between incubation period and disease severity may have a number of underlying reasons. A greater infecting dose may be associated with a shorter incubation period and increased severity. Alternatively, underlying diseases that predispose to adverse clinical outcome may also impact the incubation period and the subsequent disease severity. Since the pathogenesis of some of these diseases are related to aberrant inflammatory response, the incubation period and severity may both reflect differences in pathogenesis [7]. It has been suggested that there are differences in the underlying pathogenesis following H7N9 virus infection compared to influenza A(H5N1) virus and SARS or MERS coronavirus infections [8–10]. In this study we examined the association between the severity of human infections with H7N9 virus and the length of the incubation period.

## Materials and Methods

### Sources of data

All laboratory-confirmed human cases of avian influenza A(H7N9) virus infection were notified to the Chinese Center for Disease Control and Prevention (China CDC) and relevant clinical and epidemiological data was recorded in an electronic database [2,3]. Data extracted for this study included age, sex, geographical location, underlying conditions and dates of illness onset and hospital admission for cases reported from March 2013 through August 2014. Exposure data were available for 203/395 (51%) of cases. In the majority of those cases, the information on exposure was recorded as intervals of 2 or more days during which infection was thought to have occurred rather than exact dates of presumed infection [2]. For the subset of cases without available exposure data, we assumed their incubation time fell in the interval (0,14) days [2].

### Ethics

It was determined by the National Health and Family Planning Commission that the collection of data from A(H7N9) cases was part of a continuing public health investigation of a notifiable infectious disease and was exempt from institutional review board assessment. All patient records/information was anonymized and de-identified prior to analysis.

### Statistical analysis

A simple approach to estimate the incubation period distribution from interval-censored data is to impute the midpoint of the exposure interval for each patient, and then estimate the distribution based on these ‘exact’ incubation times. However, this approach is somewhat naïve, and is likely to overestimate incubation period distributions which tend to be right-skewed [2]. Therefore to estimate the incubation period distribution, we fitted a Weibull distribution allowing for interval censoring [2], estimating the shape and scale parameters using Markov Chain Monte Carlo in a Bayesian framework [5]. We assumed that the incubation period distribution had different parameters among the non-fatal cases and the fatal cases and we consequently estimated two different couple of parameters (k, θ) of the Weibull distribution using MCMC. To evaluate the potential factors such as age, sex, location and underlying conditions that could be associated with the length of the incubation period, we fitted to the data a multiple linear regression model using also MCMC in a Bayesian framework with the incubation period as response variable and age, sex, location and underlying conditions as explanatory variables.

To determine the association between the incubation period and the severity of disease, we first estimated the difference in mean incubation period between fatal and non-fatal cases. However this analysis could not account for potential confounders such as age that could explain a correlation, since incubation periods can vary by age [11], and severity of H7N9 infection varies by age. We therefore specified multivariable logistic regression model where death was the binary response variable and predictors included age, sex, geographical location, underlying conditions and the incubation time T_i_ of each patient [5].

We defined a logistic regression model using incubation times resampled from the 10,000 posterior samples. This approach allowed us to simulate the distribution with imputed values for individual incubation periods, which was particularly useful for an analysis in which we stratified incubation periods into tertiles.

Let *f* and *F* be the pdf and cdf of the incubation period, assumed to follow a Weibull distribution with parameters *k* and *θ* and stratified by clinical outcome (fatal and non-fatal cases).

Let *P* be the probability of death, which we assume to be dependent on age (*g*), sex (*s*), location (l), underlying conditions (c) and incubation period (*x*) as in logistic regression:

$$P(g,s,x)=\frac{1}{1+\text{exp}\left[-\left(\beta_{0}+\beta_{1}g+\beta_{2}s+\beta_{3}x+\beta_{4}l_{non-capital}+\beta_{5}l_{rural}+\beta_{6}c\right)\right]}$$ (1)

For each patient with interval-censored exposure data, we estimated 10,000 posterior samples for the incubation time using MCMC and then we estimated *θ* = (*β*_0_, *β*_1_, *β*_2_, *β*_3_, *k*_*fc*_, *θ*_*fc*_, *k*_*nfc*_, *θ*_*nfc*_) simultaneously using MCMC and the following likelihood:

$$L\left(\theta \right)=\prod_{i\in A_{1}}f(x_{i}|k_{i},\theta_{i})\prod_{i\in A_{2}}\left[F(x_{i}^{U}|k_{i},\theta_{i})-F(x_{i}^{L}|k_{i},\theta_{i})\right]\prod_{i}{q_{i}}^{d_{i}}{\left(1-q_{i}\right)}^{{1-d}_{i}}$$ (2)

where *d*_*i*_ = 1 if case *i* died from the disease and 0 otherwise; (*k*_*fc*_, *θ*_*fc*_) and (*k*_*nfc*_, *θ*_*nfc*_) are the couple of parameters of the Weibull distribution for the fatal and the non-fatal cases, respectively and where *q*_*i*_ = *P*(*g*_*i*_, *s*_*i*_, *x*_*i*_) as defined in (1).

In this analysis and the analyses described below, we specified flat priors for each parameter, and drew 10,000 samples from the posterior distributions after a burn-in of 5,000 iterations.

Analyses were conducted using R version 3.2.2 (R Foundation for Statistical Computing, Vienna, Austria). The raw data and R syntax allowing reproduction of the results presented in this article are available from the Dryad Digital Repository (link to be provided in the published version).

## Results

There were 438 confirmed H7N9 cases included in the China CDC database as of the 5th August 2014, and 43 of these were recorded as “mild” cases and were excluded from the analyses here because they form only a small sample of mild H7N9 infections. Among the 395 patients included in this study who all required hospitalization for medical reasons, 173 (44%) patients died. The mean age was 58.2 years old (±16.6 years), 71% were male, 139 (35%) cases were residents of rural areas and 189 (68%) cases had underlying medical conditions. Fatal cases were significantly older compared to non-fatal cases (63.0 vs 54.5 years old, respectively; p<0.001) and had a lower proportion of missing data on exposure (42% vs 54%, respectively; p = 0.019) (Table 1).

**Table 1:** Characteristics of H7N9 cases.

Patient characteristics	Fatal cases	Non-fatal cases	Overall	p-value*
	**All cases**			
Sample size, n (%)	173 (44%)	222 (56%)	395	-
Age (years); mean±SD	63.0 ± 15.5	54.5 ± 16.6	58.2 ± 16.6	<0.01
Male, n (%)	124 (72%)	156 (70%)	280 (71%)	0.85
Location, n (%)				0.87
Capital cities	48 (28%)	69 (31%)	117 (30%)	
Non-capital cities	66 (38%)	73 (33%)	139 (35%)	
Rural areas	59 (34%)	80 (36%)	139 (35%)	
Underlying conditions^1^, n (%)	93 (70%)	96 (67%)	189 (68%)	0.58
	**Cases with recorded exposure intervals**			
Sample size, n (%)	101 (50%)	102 (50%)	203	-
Age (years); mean±SD	60.6 ± 15.0	55.2± 17.3	57.9 ± 16.4	0.02
Male, n (%)	72 (71%)	70 (69%)	142 (70%)	0.79
Location, n (%)				0.62
Capital cities	23 (23%)	32 (31%)	55 (27%)	
Non-capital cities	40 (40%)	31 (30%)	71 (35%)	
Rural areas	38 (38%)	39 (38%)	77 (38%)	
Underlying conditions^2^, n (%)	61 (68%)	53 (65%)	114 (67%)	0.87

*p-values calculated by t-tests for age, and chi-squared tests for proportions

^1^ 119 patients had missing data regarding this information

^2^ 33 patients had missing data regarding this information

We estimated a mean incubation period of H7N9 in all cases of 3.5 days (95% credibility interval, CrI: 3.2–3.8) using a Weibull distribution. Moreover, no factors such as age, sex, location and underlying conditions were significantly associated with the length of the incubation period when fitting a multiple linear regression model (Table 2).

**Table 2:** Factors associated with the incubation period.

Factors	Coefficient β (95% CrI)^1^	Coefficient β (95% CrI)^1^
	All cases	Cases with recorded exposure dates
Age	0.007 (-0.003, 0.016)	-0. 005 (-0.019, 0.008)
Sex (male vs female)	0.158 (-0.162, 0.488)	-0.136 (-0.561, 0.293)
Location		
Non-capital cities vs capital cities	-0.040 (-0.422, 0.336)	0.131 (-0.404, 0.664)
Rural areas vs capital cities	0.016 (-0.358, 0.400)	-0.029 (-0.554, 0.497)
Underlying conditions	-0.101 (-0.530, 0.284)	-0.319 (-0.212, 0.836)

^1^ The coefficients (β) of the multiple linear regression were estimated using Markov Chain Monte Carlo (10,000 runs) with incubation period as the outcome variable and age, sex, location and underlying conditions as predictors. Moreover, 10,000 samples from the posterior distributions of the incubation periods T for each patient estimated with were used here in the multiple regression model.

**Fig 1:**
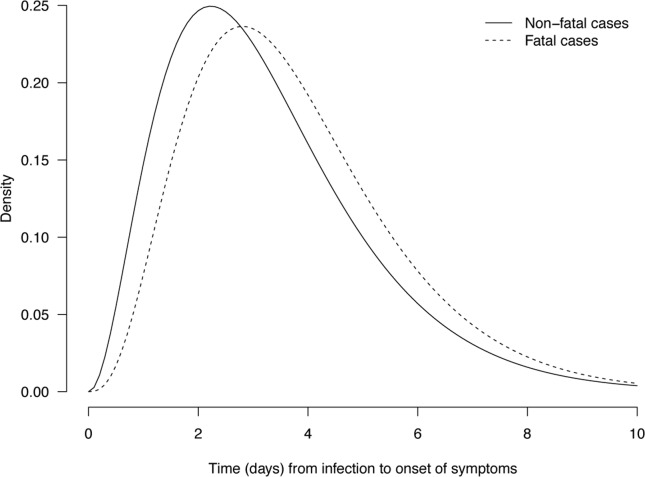
Parametric estimates of the incubation period distribution for fatal (dotted line) and non-fatal cases (solid line) of laboratory-confirmed influenza A(H7N9) virus infection. The parameters of the weibull distribution were estimated with the MCMC approach in the fatal and non-fatal cases, respectively. The estimates are for fatal cases: k = 2.30 (95% CrI: 1.80, 2.89) and θ = 4.21 (95% CrI: 3.62, 4.85) and for non-fatal cases: k = 2.03 (95% CrI: 1.62, 2.52) and θ = 3.74 (95% CrI: 3.20, 4.36).

**Fig 2:**
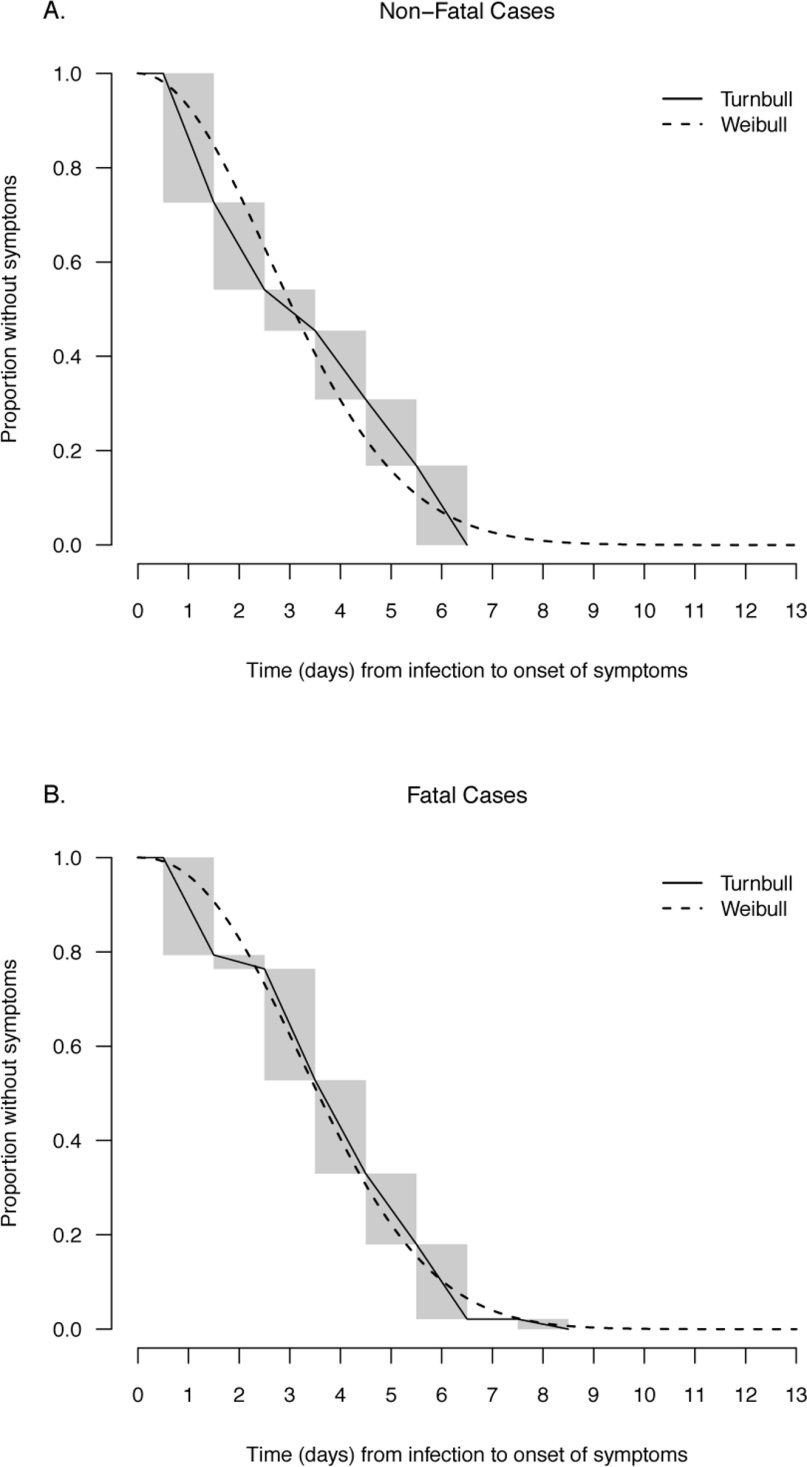
Parametric (Weibull) and nonparametric estimates (Turnbull) of the distribution of incubation periods for human avian influenza A(H7N9) virus infections for fatal cases (above) and non-fatal cases (below).

We found a longer incubation period for the 173 fatal cases with a mean of 3.7 days (95% credibility interval, CrI: 3.4–4.1) compared with a mean of 3.3 days (95% CrI: 2.9–3.6) for the 222 non-fatal cases (Fig 1 and Fig 2), and the difference in means was marginally significant at 0.47 days (95% CrI: -0.04, 0.99). In the multivariable logistic regression model, we found that a longer incubation period was associated with a statistically significant increased risk of death (adjusted odds ratio, aOR = 1.70 per day increase in incubation period; 95% credibility interval, CrI: 1.47–1.97) after adjustment for age, sex, geographical location and underlying conditions (Table 3).

**Table 3:** Factors associated with risk of death.

Factors	Risk of Death^1^OR (95% CI)	Risk of Death^1^OR (95% CI)
**Continuous incubation period using resampling method**	**All patients**	**Patients with exact exposure dates**
Incubation period^2^ (continuous)	1.70 (1.47–1.97)	1.57 (1.25–1.99)
Age in years	1.04 (1.02–1.05)	1.03 (1.01–1.04)
Sex (male vs female)	0.95 (0.58–1.55)	1.08 (0.56–2.24)
Location		
Capital cities	1.00	1.00
Non-capital cities	1.06 (0.59–1.83)	1.52 (0.68–3.42)
Rural areas	1.02 (0.58–1.75)	1.42 (0.56–3.18)
Underlying conditions	1.03 (0.57–1.78)	1.04 (0.47–2.38)
**Incubation period split into tertiles**	**All patients**	**Patients with exact exposure dates**
Incubation period^2^		
*- below 1st tertile (shortest)*^3^ *(reference group)*	1.00	1.00
*- between 1st and 2nd tertile*^3^	3.53 (2.02–6.21)	4.80 (2.16–9.73)
*- above 2nd tertile (longest)*^3^	3.90 (2.30–7.40)	7.42 (3.34–16.25)
Age in years	1.03 (1.02–1.05)	1.03 (1.01–1.05)
Sex (male vs female)	1.03 (0.61–1.69)	1.02 (0.55–1.95)
Location		
Capital cities	1.00	1.00
Non-capital cities	1.37 (0.79–2.32)	1.57 (0.71–3.66)
Rural areas	1.25 (0.75–2.16)	1.41 (0.67–3.10)
Underlying conditions	0.96 (0.55–1.67)	1.21 (0.58–2.49)

^1^ The coefficients exp(β) of the logistic regression were estimated using Markov Chain Monte Carlo (10,000 runs) with incubation period as the outcome variable and age, sex, geographical location and underlying conditions as predictors. Moreover, 10,000 samples from the posterior distributions of the incubation periods T for each patient estimated with were used here in the logistic regression model.

^2^ 10,000 samples of the incubation periods T for each patient were drawn using MCMC

^3^ the tertiles were 2.5 and 4.1 days for all patients and 2.5 and 4.2 days for patients with exact exposure dates, respectively

To examine the sensitivity of our results to inclusion of patients with imputed intervals for the cases with missing exposure data, we also fitted the logistic regression models for the subset of 203 patients with recorded exposure intervals (Table 3). We observed similar results with an increased risk of death associated with longer incubation period (aOR = 1.57 per day increase in incubation period; 95% CrI: 1.25–1.99). In addition, to examine the sensitivity of our results to the assumption of a linear association between incubation time and the log-odds of death, we categorized incubation times into tertiles and found similar results (Table 3). We also found a similar association when stratifying by age group, with point estimates of 1.35 and 2.13 in persons 0-59y and ≥60y with 95% credibility intervals including the overall estimate (Table 4).

**Table 4:** Age stratified analysis of association between risk of death and estimated incubation period, sex, location and underlying condition.

Cases	Risk of Death^1^OR (95% CI)	
	0–59 years old	≥60 years old
**All cases (n = 395)**	60/129^2^	113/93^2^
Incubation period^1^	1.35 (1.06–1.70)	2.13 (1.73–2.67)
**Cases with exact exposure dates (n = 203)**	41/57^2^	60/45^2^
Incubation period^1^	1.51 (1.10–2.03)	2.23 (1.49–3.43)

^1^ The coefficients exp(β) of the logistic regression were estimated using Markov Chain Monte Carlo (10,000 runs) with incubation period as the outcome variable and age, sex, geographical location and underlying conditions as predictors. Moreover, 10,000 samples from the posterior distributions of the incubation periods T for each patient estimated with were used here in the logistic regression model.

^2^ number of fatal cases/number of non-fatal cases

## Discussion

We estimated the incubation period of H7N9 based on cases from the first two major epidemic waves in China, and we found that fatal cases had a significantly longer incubation period than non-fatal cases. Ours is the first study to explore the potential association between the severity of H7N9 and the length of the incubation period.

In previous studies, we found that the length of incubation period in patients infected by SARS and MERS coronaviruses was also significantly correlated with the severity of the disease but in the opposite direction, with a shorter incubation period for fatal cases [5,6]. These apparently conflicting results could be a consequence of the differences in the pathogenesis of H7N9 compared to the SARS and MERS coronaviruses. SARS coronavirus evade host interferon responses but cause dysregulated pro-inflammatory cytokine responses, as also occurs in H5N1 virus infections [8,12]. Moreover, MERS-CoV and SARS-CoV present both clinical similarities and efficiently inhibit the activation of the type I IFN response which has not been shown in the case of H7N9 [13–15], although other aspects of MERS-CoV pathogenesis seems to be slightly different from SARS-CoV regarding growth characteristics, receptor usage and host response but leading finally to more severe cytopathic effects [16–18]. Recent studies showed that H7N9 induces a lower expression of pro-inflammatory cytokines, compared with the “cytokine storm” induced by H5N1 virus and SARS coronavirus [9]. H7N9 viruses also differ from H5N1 virus, SARS and MERS coronaviruses in their tropism for the human upper airways [18,19]. Thus H5N1 virus, SARS and MERS coronaviruses very likely have to directly access the lower respiratory tract and alveolar epithelium to initiate infection whereas H7N9 virus can infect the upper airways. Moreover, mortality for H7N9 cases is correlated with a wider distribution of viral antigens in the lungs [9]. Finally, human disease associated with H7N9 virus infection differs from H5N1, SARS and MERS coronaviruses in that severe H7N9 disease is associated with exacerbation of other underlying diseases, while H5N1, SARS and MERS coronaviruses cause disease in otherwise healthy persons. Collectively, these differences may explain why the fatal H7N9 cases had a longer incubation period, as the virus could spread deeper in the organism due to an abnormal immune response and potentially induced severe adverse events, such as acute respiratory distress syndrome, increasing the risk of death [9].

Moreover, we reported in this study a mean length of H7N9 incubation period of 3.5 days which is close to the mean incubation period estimated for H5N1 in other studies, with a mean length of 3.3 days (95% confidence interval, CI: 2.7–3.9) [3]. Those estimates for the mean incubation period of human infections with avian influenza viruses are longer than those reported for human infections with seasonal influenza A viruses with a median incubation period of 1.9 days (95% confidence interval, CI: 1.8–2.0), which could reflect differences regarding pathogenesis between these different virus strains [20].

We also observed a significant increased risk of death among older patients. To assess the effect of this potential cofounding factor, we applied a stratified analysis using two age groups (0–59 years old and >60 years old) and we still observed a longer incubation period among fatal cases, while the point estimate was larger among older patients (Table 4). However, these two variables were not linearly correlated in this study (Table 2). This result could reflect some potential uncontrolled cofounding effect between the length of incubation period and age, which has been reported in previous studies [5,6,11].

Our study does have some limitations. Our estimates of the incubation period were based on self-reported exposure data which could suffer from recall bias. We did not have data on the frequency or intensity of exposure, which would affect the amount of virus involved in infection. Moreover, 192 patients (49%) included in our main analysis had missing data on exposure dates, and imputation in a Bayesian framework using a wide interval of (0–14) days was necessary. However, our sensitivity analysis showed that this imputation did not affect the results in a sensitivity analysis on patients with reported exposure intervals (Table 3).

In conclusion, we found a significant association between a longer incubation period and a greater risk of death among human H7N9 cases. The underlying biological mechanisms leading to this association deserve further exploration.

## References

[1] Gao R, Cao B, Hu Y, Feng Z, Wang D, Hu W, et al Human infection with a novel avian-origin influenza A (H7N9) virus. N Engl J Med. 2013;368: 1888–1897. 10.1056/NEJMoa1304459 23577628

[2] Virlogeux V, Li M, Tsang TK, Feng L, Fang VJ, Jiang H, et al Estimating the Distribution of the Incubation Periods of Human Avian Influenza A(H7N9) Virus Infections. Am J Epidemiol. 2015;182: 723–729. 10.1093/aje/kwv115 26409239 PMC4597801

[3] Cowling BJ, Jin L, Lau EHY, Liao Q, Wu P, Jiang H, et al Comparative epidemiology of human infections with avian influenza A H7N9 and H5N1 viruses in China: a population-based study of laboratory-confirmed cases. Lancet Lond Engl. 2013;382: 129–137. 10.1016/S0140-6736(13)61171-X PMC3777567 23803488

[4] Moser MR, Bender TR, Margolis HS, Noble GR, Kendal AP, Ritter DG. An outbreak of influenza aboard a commercial airliner. Am J Epidemiol. 1979;110: 1–6. 463858 10.1093/oxfordjournals.aje.a112781

[5] Virlogeux V, Fang VJ, Wu JT, Ho L-M, Peiris JSM, Leung GM, et al Brief Report: Incubation Period Duration and Severity of Clinical Disease Following Severe Acute Respiratory Syndrome Coronavirus Infection. Epidemiol Camb Mass. 2015;26: 666–669. 10.1097/EDE.0000000000000339 PMC4889459 26133021

[6] Virlogeux V, Park M, Wu JT, Cowling BJ. Association between severity of MERS-CoV infection and incubation period. Emerg Infect Dis. 2016;In Press. 10.3201/eid2203.151437 PMC4766874 26890291

[7] Ho M-S, Chen W-J, Chen H-Y, Lin S-F, Wang M-C, Di J, et al Neutralizing antibody response and SARS severity. Emerg Infect Dis. 2005;11: 1730–1737. 10.3201/eid1111.040659 16318725 PMC3367364

[8] Peiris M. Pathogenesis of avian flu H5N1 and SARS. Novartis Found Symp. 2006;279: 56–60; discussion 60–65, 216–219. 17278385

[9] Meliopoulos VA, Karlsson EA, Kercher L, Cline T, Freiden P, Duan S, et al Human H7N9 and H5N1 Influenza Viruses Differ in Induction of Cytokines and Tissue Tropism. J Virol. 2014;88: 12982–12991. 10.1128/JVI.01571-14 25210188 PMC4249090

[10] Hui DS, Memish ZA, Zumla A. Severe acute respiratory syndrome vs. the Middle East respiratory syndrome. Curr Opin Pulm Med. 2014;20: 233–241. 10.1097/MCP.0000000000000046 24626235

[11] Cowling BJ, Muller MP, Wong IOL, Ho L-M, Louie M, McGeer A, et al Alternative methods of estimating an incubation distribution: examples from severe acute respiratory syndrome. Epidemiology. 2007;18: 253–259. 10.1097/01.ede.0000254660.07942.fb 17235210

[12] de Jong MD, Simmons CP, Thanh TT, Hien VM, Smith GJD, Chau TNB, et al Fatal outcome of human influenza A (H5N1) is associated with high viral load and hypercytokinemia. Nat Med. 2006;12: 1203–1207. 10.1038/nm1477 16964257 PMC4333202

[13] Zielecki F, Weber M, Eickmann M, Spiegelberg L, Zaki AM, Matrosovich M, et al Human cell tropism and innate immune system interactions of human respiratory coronavirus EMC compared to those of severe acute respiratory syndrome coronavirus. J Virol. 2013;87: 5300–5304. 10.1128/JVI.03496-12 23449793 PMC3624328

[14] Kindler E, Jónsdóttir HR, Muth D, Hamming OJ, Hartmann R, Rodriguez R, et al Efficient replication of the novel human betacoronavirus EMC on primary human epithelium highlights its zoonotic potential. mBio. 2013;4: e00611–00612. 10.1128/mBio.00611-12 PMC3573664 23422412

[15] Kopecky-Bromberg SA, Martínez-Sobrido L, Frieman M, Baric RA, Palese P. Severe acute respiratory syndrome coronavirus open reading frame (ORF) 3b, ORF 6, and nucleocapsid proteins function as interferon antagonists. J Virol. 2007;81: 548–557. 10.1128/JVI.01782-06 17108024 PMC1797484

[16] de Wilde AH, Raj VS, Oudshoorn D, Bestebroer TM, van Nieuwkoop S, Limpens RWAL, et al MERS-coronavirus replication induces severe in vitro cytopathology and is strongly inhibited by cyclosporin A or interferon-α treatment. J Gen Virol. 2013;94: 1749–1760. 10.1099/vir.0.052910-0 23620378 PMC3749523

[17] Josset L, Menachery VD, Gralinski LE, Agnihothram S, Sova P, Carter VS, et al Cell host response to infection with novel human coronavirus EMC predicts potential antivirals and important differences with SARS coronavirus. mBio. 2013;4: e00165–00113. 10.1128/mBio.00165-13 23631916 PMC3663187

[18] van den Brand JMA, Smits SL, Haagmans BL. Pathogenesis of Middle East respiratory syndrome coronavirus. J Pathol. 2015;235: 175–184. 10.1002/path.4458 25294366 PMC7167882

[19] Chen Y, Liang W, Yang S, Wu N, Gao H, Sheng J, et al Human infections with the emerging avian influenza A H7N9 virus from wet market poultry: clinical analysis and characterisation of viral genome. The Lancet. 2013;381: 1916–1925. 10.1016/S0140-6736(13)60903-4 PMC7134567 23623390

[20] Lessler J, Reich NG, Brookmeyer R, Perl TM, Nelson KE, Cummings DAT. Incubation periods of acute respiratory viral infections: a systematic review. Lancet Infect Dis. 2009;9: 291–300. 10.1016/S1473-3099(09)70069-6 19393959 PMC4327893

